# Collectanea

**Published:** 1830-07

**Authors:** 


### COLLECTANEA.

Floriferis ut apes in saltibus omnia libant,
Omnia nos, itidem, depascimur aurea dicta.

### PATHOLOGY.

*Disease from the use of Iodine, and the Disorders which arise from the long-continued Employment of that Remedy.* By Dr. JAHN. (*Journ. Comp.*)

SINCE the researches of ORFILA, the injurious effects which arise from too large a dose of iodine are well known. The great irritation produced by the vapours of this substance, both upon the eyes and aerial passages, is also proved:* but there is one point upon which there still exists some diversity of opinion, namely, the changes effected upon the different tissues by the long-continued use of preparations of iodine. COINDET, GAIRDNER, SEILER, and others, have given descriptions of a cachetic condition produced by this remedy, which does not coincide with the experience of Dr. JAHN; and they have explained the facts they have observed in a very different manner from the last-named physician. The following is the description he gives of the iodine disease.

When introduced into the organic fluids, iodine acts firstly and principally upon the process of nutrition. The first evident effect is an absorption of the fat, so that a gradual leanness is remarked. At the same time we may observe, with a little attention, an augmentation of all the excretions. The skin, in consequence of an increased deposition of carbon upon it, appears dull and of a livid hue. There is great and clammy perspiration. Respiration is obstructed. The urine is increased in quantity, and the surface of it is often covered with an oily pellicle. The alvine evacuations are increased,

* We have already referred to a case in our own practice, in which temporary loss of sight, giddiness, &c. arose from the use of the iodine ointment. —EDITOR.

and the feces are loaded with bilious matter, and contain but little mucus. The seminal secretion is increased, and also the menstrual discharge. It is clear, says M. Jahn, that, in this state, the vitality of the veins and lymphatic vessels is exalted, and the predominance of venous excitement is shown by the swollen state of the superficial veins and the blue colour of the lips. The blood, as may be inferred from the diminished redness of the skin and the feebleness of the arterial pulsations, has acquired a more serous character, and is more liquified; so that the quantity of serum is greater in proportion to the cruor and fibrine. The energy of the irritable tissues is comparatively diminished. Hence the patient is more easily fatigued than before; digestion is irregular; the saliva and mucus are diminished, and complaint is made of dryness of the mouth and throat. The nervous power is also materially affected, and symptoms resembling hysteria and hypochondriasis arise; morbid sensibility, lowness of spirits, timidity, sensation of weakness, trembling of the limbs similar to that produced by mercury, agitated sleep, with disagreeable dreams, &c. At this period, irregular and transient febrile attacks announce a reaction of the constitution. If now the morbid condition be not opposed, and if the iodine be continued, the above symptoms increase in severity; and shortly the glandular tissues, the breasts, testicles, and thyroid gland, are diminished in substance. At length all those symptoms arise which are said to constitute nervous consumption.

M. Jahn has examined two bodies, which presented the traces of the action of iodine. A woman, who, having misused the remedy, was attacked with enteritis, which proved fatal; and a man, affected with cancer of the stomach, who was treated by the internal and external use of iodine, and who took very large doses of the tincture secretly, in hopes of a more speedy cure. In the bodies of these patients, the fat had disappeared, the various tissues had a withered and flabby appearance, the glands were shrunk and soft, and also the mesenteric ganglia, (which are usually much developed in cancer of the stomach,) the thyroid and supra-renal glands, the liver, spleen, and ovaries.

Notwithstanding the mischief sometimes inflicted by the use of iodine, M. Jahn considers it one of the most valuable remedies which have been recently discovered.

---

*Case of Gonorrhœa, alternating in a singular manner with Bilious Remittent Fever.* By Lewis Campbell, M.D. of Moutton, Alabama.

On the 23d of July, 1827, I was called by Mr. ——, who had been affected with a gonorrhœa three ——— since; and, as he had been obliged to ride some distance every day on business, the symptoms were somewhat aggravated. He suffered greatly from ardor urinæ and chordee. The discharge of matter from the urethra was very profuse, and considerable general excitement was present. His bowels were freely opened with sulphas magnesiæ; one drachm of balsam of copaiva was directed to be taken three times a day, and a low diet enjoined. He obtained sudden relief from the ardor urinæ from this course; but a profuse discharge of matter, and the retraction of the urethra remained. His business calling him from home, he was under the necessity of riding some distance every day till the 26th, when he was attacked with a bilious remittent fever. During the evening he felt languid, sick at stomach, &c. At eight in the evening he was taken with a violent chill, followed by fever.

During the chill the gonorrhœal discharge was suddenly suppressed. On the morning on the 27th, I found him entirely free from all the symptoms of gonorrhœa. He assured me that he felt no inconvenience from that source.

The fever continued till the 30th. During this time he enjoyed entire exemption from the gonorrhœal symptoms. On the 30th, his general health being much improved since the preceding day, he escaped fever, but he was again attacked with gonorrhœa, and chordee. He was again put under treatment for those affections. His general health continued to improve till the 2d of August, when, from exposing himself to the sun on a short ride in the heat of the day, the bilious symptoms returned; and he then had chills and fevers two days in succession. During the first chill the running was again suppressed; and he was completely relieved from any uneasiness in the urethra during the continuance of the febrile symptoms. On the 4th he experienced exemption from fever and a return of gonorrhœa. His general health continued to improve from this period, though the affections of the urethra proved somewhat lingering. His stomach rejected the balsam in any form, and scarcely tolerated the presence of any medical substance whatever. Regimen and time affected a cure. Was the suppression of the gonorrhœa during the fever, and its immediate return when the fever yielded, a mere coincidence? Or was it a metastasis of morbid action from the urethra to the stomach, and from the stomach to the urethra? If the latter, does it not favor the opinion of the physiological school relative to the non-specific nature of venereal affections?—*North American Med. and Surg. Journal.*

---

*Cases of Pneumonic Inflammation, attended with copious Expectoration of Pus, and ending in Recovery.* By Dr. F. Bache.

Case I. William Coulter, a mulatto labourer, aged twenty-two years, was admitted into the hospital in the month of March 1828. He had been treated for pneumonic inflammation at the Arch street prison, before his transfer to this prison, and was still sick when he arrived. Up to the 1st of April, he had been bled and blistered twice, and took antimoniated barley-water and decoction of seneka. He has expectorated pus freely for about three weeks, and since the free expectoration has been better. At present (April 1st,) the expectoration is somewhat diminished, and the patient is getting better. The appetite is good, sleep easy, bowels open, skin cool, pulse regular.—Ordered the decoction of seneka to be continued, and a nourishing diet.

April 3d.—Cough rather more frequent; appetite continues good; expectoration the same. Continue the seneka, and take, in addition, the liquorice mixture.

5th.—Patient feels pretty well; expectoration inconsiderable. A puriform liquid of a bad smell is discharged from the nose. Breast without pain, bowels open. Pronounced to be convalescent. Continue the same remedies.

8th.—Expectoration same. Matter from the nose not so fetid. Had a griping yesterday, and took today half a drachm of rhubarb. Pulse good; appetite very good; sleep natural. Omit seneka, and continue the liquorice mixture.

10th.—The patient says he feels well, though he still expectorates. Discharged relieved.

July 23d.—Today I met Coulter in the street; he said he was quite well.

*Remarks.* I have copied this case just as it stands in my note book, where I entered it as a case of phthisis. It had the aspect of that disease, being characterized by emaciation and copious purulent expectoration. Still the result proved it to be one of those cases in which the lungs are not deeply implicated. Unquestionably the pus in this case came from abraded and inflamed surfaces, and not from abscesses or suppurating tubercles. I am thoroughly satisfied that, when suppurating tubercles exist, we have not yet learned how to arrest the disease; and I have reason to believe that it is such cases as the one here reported that pass for instances of cured phthisis. It is not always easy to distinguish the true from the spurious cases, but I should be disposed to say, as a general rule, that when the pulse is quick, and dyspeptic symptoms are present, associated with cough and purulent expectoration, the fatal disease, which is understood by the name of pulmonary consumption, may be considered as fully formed.

Case II. John Hartman, farmer, aged twenty-four years. This also was a case of pneumonic inflammation, ending in suppuration. The subject of it entered the hospital on the 5th of February, 1828, and was for some time very ill. A free expectoration of pus came on, with a subsidence of all the bad symptoms. About the 25th of March he became worse, being affected with pain in the chest, hard cough, and fever; and the expectoration becoming less free, I ordered a blister to the breast, and the sweet spirit of nitre, and discontinued some tonic medicines which he had been previously taking.

April 1st.—This is the fifty-sixth day of his disease, and he takes seneka decoction and an anodyne mixture at night. His diet consists principally of milk, bread, and chocolate. The patient gets slowly better, and I entertain hopes of his recovering, from the great developement of his chest.

3d.—Patient feels weak and feeble; no pain; appetite moderate; sleep pretty good the previous night; bowels open.—Omit the anodyne mixture, continue the seneka. Apply one grain of morphia to a small blister on the chest.

5th.—Tried the morphia by the endermic method last night, but without effect. Cough straining; expectoration diminished and difficult.—Apply two grains of morphia to the blistered surface.

8th.—Has slept better the three last nights from the use of the morphia. The cough is less straining; and the matter, which is in small masses, is expectorated with greater facility.—Continue seneka and morphia.

10th.—Rested well the two last nights, with the exception that he sometimes awakes in a fright. Coughs and expectorates easier. The expectoration consists of ropy pus and mucus. Bowels open; appetite moderate; debility considerable.—Continue seneka and morphia. Ordered mutton daily.

12th.—Slept badly last night. The morphia being expended, none was applied last evening. The expectoration is diminished and more difficult; no sweats.—Continue the seneka.

15th.—Has not rested so well since the external application of the morphia was discontinued. Thinks his cough is worse.—Continue the seneka; substitute two grains of opium by the mouth for the morphia.

29th.—The patient continued to improve up to this date, the eighty-fifth day of the disease, when he was so far recovered as to be discharged from the hospital.

On the 25th of June this patient returned to the hospital, with universal pains and spitting of blood. He was bled, cupped, took several Dover's powders, and was discharged cured in four days.

*Remarks.* This case was somewhat like the last, inasmuch as the first symptoms led me to suppose that the lungs were affected, as they generally are in fatal consumption, that is, with tubercles. The prognosis, however, was more favorable, on account of the age of the patient (forty-four years,) and the great capacity of his chest. The morphia, employed by the endermic method, seemed to act favorably as an expectorant, and lessened the sufferings of the patient. It did not seem to disorder the stomach; and if this should be found generally true, the endermic employment of morphia will be a great resource where the different preparations of opium disagree with the stomach.—*Ibid.*

---

*Caries of the Alveolar Process of the Upper Jaw, with the Reproduction of the Jaw Teeth after its Cure.* The following case is related in Rust's Magazine, b. 29, h. 2, 1829.

H. S., a female, thirteen years of age, who had suffered repeated attacks of scrofulous disease, which, though somewhat obstinate, yielded at length to medical treatment, was seized with severe pain of the left portion of the upper jaw, affecting also the teeth of the same side. The left cheek became swollen, and a hard tumor appeared upon the gum, of the size of a hazel nut. The disease being supposed to be simply a gum bile, no professional aid was procured for fourteen days. During this time the alveolar arch, internally, was protruded in such a manner as to form a convex swelling within the mouth. Dr. Samuel, of Conitz, was now requested to see the patient. He concluded it to be a case of suppuration of the antrum; which diagnosis was strengthened by the circumstance of the patient discharging, in sneezing, from the left nostril, a fetid purulent matter. The third molar tooth was extracted, and the bottom of its socket was pierced with a trochar; a quantity of putrid pus was discharged. The perforation was kept open by means of a small plug, which was subsequently gradually enlarged. Into the antrum was injected a decoction of bark, with honey of roses and tincture of myrrh, part of which passed out at the left nostril. The first, second, and fourth molar teeth becoming loose, were removed at different periods. Soon afterwards a portion of loosened bone presented itself at the opening made into the antrum, which was extracted, the opening being first enlarged. It was about the size of a silver groschen, and of an irregular square shape. A similar portion of bone was subsequently extracted. The cure was effected in about six months by means of the foregoing injection and the administration of Rubia tinctorum and bark. The jaw, with its alveolar process, resumed its normal shape, and within the first year after the cure other teeth were cut, in the place of those which had been removed.

---

*Morbid Appearances in the Brain, after frequent attacks of Apoplexy.* The wife of a labourer, forty-one years of age, of long-continued intemperate habits, had experienced repeated attacks of apoplexy, after the last of which she had remained imbecile, and subject to occasional fits of epilepsy. During an attack of the latter she died suddenly. On opening the head, the bones of the cranium were found to be extremely hard, and of unusual thickness. The brain was of medium size, free from congestion, and of natural consistency.

On cutting into the substance of the organ, there were discovered, at the external part of the medullary portion of the right hemisphere, on a level with the thalamus nervi optici, two cavities, of the size of a small bean, and filled with a clear fluid. They were both surrounded by a coat of firm consistency, similar to a serous membrane; this was intimately connected by its exterior portion with the surrounding substance of the brain: each could, however, be dissected out, in the form of a complete sac. Their internal structure was cellular. Near to these, but more internal, and higher up, there was found a grayish or red gelatinous mass, very distinct from the medulla of the brain: this had already begun to be surrounded by a covering similar to the former. The ventricles were loaded with water. The pineal gland contained a large sand-like concretion. A considerable amount of fluid escaped from the spinal cavity. The viscera of the thorax and abdomen were sound; the stomach was, however, contracted to the size of the duodenum.

This case is related in Rust's Magazine, band xxx. heft 1, 1829.

---

*Case of Congestion of the Cerebellum, with involuntary walking backwards.* The Chevalier D., a widower, about fifty-six years of age, of a sanguine temperament and a good constitution, was cured of a pleuro-bronchitis. Two months afterwards, on the 9th of November, he was attacked with a pharyngitis; which was combated by an application of twenty leeches to the upper and lateral parts of the neck, by emollient cataplasms, mucilaginous drinks, &c. On the evening of the 13th, he was affected with a strong flush in the face, particularly over the cheek bones; eyes brilliant, and slightly injected; pulse strong, but little increased in frequency; skin hot; sensation of distress in the occipital region, and difficulty in performing the lateral movements of the neck. In the night occurred vertigo, giddiness, and an obtuse pain in the occiput. M. D. went on his knees to evacuate the urinary bladder, and was in great danger of falling backwards; which he prevented by grasping the head of his bed. Continual and fatiguing erections continued the whole night, but without emissions. Next morning, the injection of the face had disappeared, but a similar appearance had taken place in the occiput and back of the neck, with a slight tumefaction, but no pain on pressure; pulse the same as before. M. D. experienced giddiness, but walked a distance of about two gunshots: he was obliged to stop several times, to avoid a fall backwards, and to resist the tendency he felt to walk in that direction. He even made a step backwards, which he designed for the forward direction. When arrived at his place of destination, he again experienced giddiness, and a fresh propensity to walk backwards. He made nine or ten steps backwards, and would have fallen on his back if he had not supported himself upon a piece of furniture. The same propensity continued till his return home; when the symptoms immediately gave way before a bleeding of eighteen ounces, low diet, acidulated drinks, and a footbath with mustard. Next morning the Chevalier was quite well.—*Journal de Physiologie.*

---

### PRACTICAL MEDICINE.

*Syphilis cured in an Infant by Mercurial Frictions applied to a Goat that suckled it. (Archives,* Avril.)

Dr. Veré Delisle lately communicated this case to the Académie Royale de Médecine. A woman, three months after delivery, who suckled her infant, contracted a syphilitic disease, characterized by ulcerations on the inside of the labiæ and a gonorrhœal discharge. The child was soon affected with venereal pustules and ulcerations around the margin of the anus. It was now suckled by a goat; and, after the inside of the thighs of the animal were shaven, two drachms of mercurial ointment were rubbed in each other day. The child was cured in a month.

### SURGERY.

*Remarks on Superficial Cancers, with Cases in which the Patients were cured without the amputation of important Organs.* By M. Lisfranc. Read at the Academy of Sciences of Paris.

The object of the author of this memoir is to prove that the surgeon may frequently save either a part or the whole of an organ, in cases which have been before considered to require the complete removal of it. Recent discoveries in pathological anatomy have shown that cancerous diseases do not, at the same time, invade all the tissues of the organs attacked. For example, in cancer of the stomach, the disease is sometimes limited to the muscular tunic, or to the cellular substance which unites it to the mucous membrane, and when all these parts are affected with the disease, we may detect, by a careful dissection, the particular part in which the malady originated. This progressive succession in the march of cancerous diseases has for a long time attracted the attention of M. Lisfranc, in his examination of the bodies of patients who had died with cancer of the breast. He ascertained, by the most attentive investigation, that the disease had for years been arrested by the pleura, which remained untouched in the midst of the malady which surrounded it. In three subjects which had died with old carcinoma of the umbilicus, he remarked that the peritoneum offered the same opposition in the abdomen as the pleura in the thorax, to the extension of the disease. The same fact was observed in various cases, in which the parts attacked with cancer were contiguous to cavernous bodies.

In reflecting upon these facts, M. Lisfranc conceived the possibility of turning to the advantage of surgery the evidence afforded by pathological anatomy. Having observed that, in the majority of cases, cancer is confined to one tissue, he inferred that it might be necessary to remove only the part diseased, and not the whole organ. Experience soon proved that this idea was correctly formed, and many operations conducted upon this principle were crowned with the most complete success.

In two cases of cancer of the penis, the patients were saved from the most melancholy of all surgical mutilations. In the third case, the patient was attacked with cancer of the tongue. The two right thirds of the organ were diseased, and were hard, tumefied, and ulcerated; the whole substance being affected. Many of the most distinguished surgeons of Paris had seen the patient, and all of them had advised the total extirpation of the two thirds which were diseased. The healthy parts were separated from the diseased parts with a bistoury, and the latter were surrounded with a ligature, which was drawn moderately tight. No bad symptoms followed, and during the succeeding six days the tightness of the ligature was gradually increased: the portion included within it shrunk, became black, and fell off. The natural breadth and length of the tongue were still preserved, with the exception of a very small portion of the tip. The superfices had alone been diseased, and that alone was sacrificed by the operation. The parts beneath remained, and cicatrized under the influence of emollient and resolvent applications. A small ulcer remained for some time, but yielded to cauterization with the nitrate of silver. Several months afterwards the patient was shown to the Academy: he was perfectly cured, and enabled to resume his business of an advocate, in the exercise of which his tongue was, of course, a most important part.

M. Lisfranc, in concluding his memoir, draws the following conclusions:

1. That, whatever may be the ravages inflicted upon the organic tissues by cancer, nature tends to limit the extent of the disease.

2. That morbid anatomy having afforded probable evidence of the nature of these limits, we may hope to save the organs affected, by removing only the tissues which are diseased.

3. That this idea, derived from the progress of pathological anatomy, has been acted upon with success in the cases related, and in many others which will be submitted to the profession.

That we sincerely hope the opinions of M. Lisfranc may prove to be well founded, will not be doubted; but, upon a subject of such vast importance, the clearest and most satisfactory evidence will be required. It will be necessary to instruct us how we are to ascertain the extent of cancerous disease. By what means shall we determine that one component tissue of an organ is affected with the malady, and that another is not. M. Lisfranc has formed his opinion of the limits which nature assigns to cancer by dissection; but the question is not how we may establish this important fact in the dead body, but how we may discover it in the living, so that we may adapt our practice to the extent of the disease. If, however, M. Lisfranc had only thrown out a hint upon the subject, totally unsupported by practical experience, it would demand the attention of the profession; but he comes before us with stronger claims, the successful result of his practice. We earnestly wish that other surgical observers may confirm his views, and that the partial removal of a cancerous organ may be found to be safe and justifiable. At present we confess we are not satisfied upon this point, although we are much gratified at the distant prospect M. Lisfranc presents to us of the possibility of lessening the severity of many dreadful surgical operations.

---

*Nævi Materni.* (From Mr. LAWRENCE'S *Lectures on Surgery.*)

Under the name of Nevi Materni are included various original deformities or peculiarities of the structure of some portion of the skin. The name nævi materni, which means simply mother's spots, is founded on the generally received notion that those peculiarities in the skin arise from some influence of the mind of the mother on the offspring. It has been supposed, for instance, that if a pregnant woman is terribly frightened by any strange sight, that the skin of the child will probably bear some mark on the body more or less allied to the object that has produced this terror. Or, again, if a pregnant woman should take a strong desire, or, as they sometimes call it, a longing for any thing, especially if the longing cannot be gratified, there is an idea that this object will be marked upon the child. Thus, if we may believe mothers and nurses, the objects that I am now going to speak of are only so many representations of things, whether of food or any thing else, that the mother has been longing for; whether it be beef or pork, raspberries or grapes, or the

Lord knows what. This idea seems to be very prevalent; the same appearances are called in French *envies*, which means mother's longings: the name they bear in German is the same, *mutter-mahl*, mother's spots.

We sometimes find an elevation of the skin, an irregular figure, of a rough granular surface, generally reddish, or brownish, or yellow, but varying in point of colour, and not uncommonly having on it particularly long hairs. These have been called in common language, *moles;* and they generally remain throughout life of their original size; they do not increase. It might happen that a mark of this kind was so situated as to produce a deformity; and if it is necessary, you can remove it, the operation of extirpation being a very simple one. There are some instances of these elevations of the skin growing after birth, and attaining a large size, of which this specimen is an example.

[Mr. Lawrence here represented a preparation, observing, "This was cut from the lower part of the back of a lady, then an adult. It had been a 'mark' originally, and I do not know that I can give it any other name than that of a huge mole. It measures about one foot in length, and is about one foot in breadth. It had a bright red appearance during life. It was removed by Mr. Abernethy, and I assisted him in the operation, many years ago. It did not go deeper than the skin: this is the adipose substance under it."]

Sometimes a part of the skin is brownish or reddish, and thickly covered with hairs, like the coat of an animal. A little time ago, I remember there was a boy in the foul wards. I had him stripped to examine some syphilitic eruption. There was a part on the chest, about two inches one way and one inch the other, of light brown hair, like the coat of an animal. I looked at it, and said, "What is that on the chest?" "Oh, sir," said he, "it is a mouse." I said, "it is not a mouse; it has no tail." "Ah," said he again, "it had a tail, but somebody cut it off." I saw a curious looking child, a little time ago; a gentlemen sent for me to see it as a curiosity. I think that a great part of the body was covered with hair. Over the lower extremities there was a great number of spots; one on the arm, and others on the trunk of the body. The skin covering the child, in other respects, was of the natural texture, and, indeed, these parts were not unhealthy.

In the greater number of these nævi, however, there is an unnatural state, particularly of the vessels of the skin. This peculiarity seems to consist more or less in the vascular tissue of the part. Sometimes you have a few vessels enlarged and ramifying superficially in the skin; sometimes giving not an inelegant kind of appearance. This has been called *spider nævus*, the ramifications bearing some analogy to the legs of a spider. Not uncommonly, you see parts of the skin discoloured, red, brown, or livid; sometimes of a deep tint, sometimes lighter, and this extending irregularly over a considerable part of the skin; very frequently on the face. These are commonly called *claret marks*, the colour being something like the stain produced by claret. You often see large vessels ramifying quite on the surface of the skin, and in some instances of that kind I have known individuals liable to occasional bursting of these vessels, and to very copious hæmorrhage from them. But these marks I am now speaking of generally are of a *stationary* kind; they do not increase; they remain in the same state through life. The nævi which are most commonly the subjects of surgical treatment, consist of peculiar vascular growths, seated either in the skin itself, or in the adipose tissue immediately under it. The *cutaneous nævi*, or those seated in the skin, consist of a soft bright scarlet elevation, the surface of which is finely granulated. They appear to occupy a certain portion of the texture of the skin, as if a part of it were stained with this bright scarlet colour. It is really rather an elegant sort of texture which occupies the place of the natural structure of the skin; it is generally of a more or less circular figure, not rising high above the natural elevation of the skin. The subcutaneous nævi consist of soft swellings seated under the skin, imbedded in the adipose tissue, the skin itself being completely sound over them. Towards the centre of the nævus, you have more or less of a blue or livid appearance. It seems as if some of the vessels were running out of the nævus to approach nearer the surface of the skin in the centre, so that you have a blue, or livid, or black discoloration in the centre, and a tumor extending in circumference under and much beyond the sound skin, in the cellular membrane beneath. This is quite a soft tumor. Not unfrequently you have a portion of cutaneous nævus in the centre, and part of a *subcutaneous* tumor extending under it: so that, when we speak of cutaneous and subcutaneous nævi, you are not to suppose that they are so essentially different in their nature that they cannot be combined together. You have in these nævi one part which is a bright scarlet soft state of the skin, and another which forms a tumor under it. Both these kinds of swellings are soft and compressible; in fact, if you press upon them, you find that you diminish their bulk; that you squeeze something out of them; and when you remove the pressure, they slowly recover their former size. Sometimes they are rather firmer than the rest of the skin, and there is a sensible increase of heat in them.

These nævi, like the others that I have mentioned to you, may remain stationary; that is, you see them of a certain size at the birth of a child, or they acquire a certain size soon after, and then do not grow further. More commonly, however, they are small at the time of birth, and begin to increase, and often grow very rapidly for some time. With respect to those that are permanent, they do not always remain in the same condition. There are certain changes, according to the state of the system and the particular circumstances affecting the individual, so that growths of this kind will sometimes be more full and tense, the vessels being apparently more filled; at another time they are paler and more flaccid. There are the cutaneous nævi, which are sometimes compared to strawberries, raspberries, and so forth; and really the simile is not altogether inapt. I have many times been seriously told, and that by well-informed persons, that a certain mark on their daughter's back, which they called a raspberry, increases at the time that fruit comes in, and gets larger and generally redder by the time that raspberries are ripe. It then withers, shrinks, and disappears every year. It is these changes in the state of the nævus which have given rise to, and may support, this notion.

I mentioned to you that these nævi are usually small at the time of birth, and often perhaps, so trifling as to be overlooked till some time after. Then they begin to increase in size, and grow rapidly. Thus you may see them of the size of a pin's head, or of the whole hand; indeed, there is hardly any limit to their growth. They are most common on the head; frequently they are found on the scalp, and upon all parts of the face; and sometimes they seem to occupy nearly the whole of a part; such as the ear, the eyelid, or the lip. I remember seeing the case of a young woman, at this hospital, where half of the lip, and the corresponding part of the side of the face, seemed to be entirely filled with this unnatural growth. It was covered by the skin externally, and by the mucous membrane of the mouth internally. These nævi are sometimes seated about the neck, often extending deeply; they are more rarely seated upon the extremities of the body; sometimes they are found upon the trunk.

Those that grow consist essentially of large vessels near the surface, and it will happen frequently that these vessels will give way. They are liable to be ruptured by accident, and thus profuse hæmorrhage will take place, or sometimes, after attaining a certain size, a state of ulceration and inflammation comes on, and this ulceration, if it become extensive, will frequently destroy a considerable part of the morbid growth, and lead to a kind of partial natural cure of the affection. I have mentioned to you that pressure will diminish the size of the nævi, and that they recover their former magnitude when the pressure is removed. This effect is produced by squeezing the blood out of them; for what they contain, which is thus removed by pressure, is blood; and moreover it is arterial blood. In the cutaneous nævus, which is quite on the surface of the body, and where the vessels are only covered by a thin kind of cuticular integument, you have a bright scarlet tint, showing that it is arterial blood. If the blood be in a deeper seated tumor, with a great thickness of integument over it, the colour is blue or livid, so that you might suppose that it was produced by venous blood; but you find, if you come to cut into such nævi in operations, that it is arterial blood which flows from them. I may mention to you, which is a further evidence of what they contain, that if an incision be made in operating, if you cut into any part of one of these growths, arterial blood flows out in a quantity and with a degree of violence that you can hardly imagine; and which you would scarcely believe possible, unless you saw it. A copious stream of the most florid arterial blood comes out, and you cannot restrain it unless you press firmly on the whole of the surface that is injured by the incision.

When you come to make a section of a tumor of this kind, after removing it, it exhibits a cellular appearance, and the general notion has been that it is made of cells, with large vessels ramifying through, and depositing blood in these cells. Now I believe this notion of the cellular structure of these nævi is not a correct one. So far as I have observed when a section is made, the apertures that are seen are the mouths of blood-vessels; they are all regularly circular, with a smooth lining, like that of the blood-vessels. They appear to me, therefore, to consist essentially of an aggregation of vessels ramifying and combining together into a kind of tumor. Whether they be arteries or veins, I do not undertake to determine, but I can only say that during life they appear to contain arterial blood, and when you cut into them, florid blood flows in the greatest profusion. Hence the name that is sometimes popularly given is by no means a bad one, *bloody tumors*; and they are described by Boyer under the title *tumeures variqueuses*, which is the same as our popular term, and both of which designate that they depend materially on blood-vessels. Dupuytren has turned his attention to the structure of these nævi, and his idea is, that they consist of the same kind of texture which is found naturally in certain parts of the body. Thus there are certain parts in which you find that the structures are usually flaccid, but which admit of distention by an increased influx of blood; they admit of the state which has been called *erection*, and the structure has been called by the French *tissue erectile*, erectile tissue, like that of the penis, the clitoris, the nipple of the female, and some other parts. Dupuytren considers that these nævi are unnatural productions of erectile tissue in the parts in which they are found. Hence he has called them *tumeures erectiles.* There are some points of analogy between these nævi and the erectile tissue, but I must observe that we do not know quite enough of either of these two tissues thus compared, to be prepared to assert or to deny their identity. I would observe, however, that this kind of structure not only exists as a congenital production, but may take place as an accidental formation after birth in various parts of the body. Under such circumstances, the tumor, although perhaps not precisely corresponding to the description that I have given to you, has, in many cases, another character, which is very important; that of pulsation, having a pulsation synchronously with that of the arteries. This is a circumstance which I have not observed in nævi materni, that is, in the congenital kind, and certainly does not exist in the greater number of them, though it has been sometimes observed. Dupuytren has alluded to it; he speaks of these tumors generally as having some pulsation. This is the affection which John Bell calls aneurism by anastomosis, and others have done the same after him. Hence it has been supposed that the congenital marks or nævi are of this nature; but as I mentioned to you, they do not possess that particular feature upon which Mr. Bell founded his opinion, namely pulsation, nor can these same growths be correctly denominated aneurism, for there is no kind of analogy between vessels inosculating with each other as these do, and that affection of the arterial trunks which we call aneurism.

The same principles of *treatment* are applicable to this affection, whether it exists congenitally, as nævus maternus, or whether it is an accidental production taking place subsequent to birth.

If nævi materni be stationary; if they do not increase in size, there can be no reason for interfering or meddling with them, unless they be so seated as to be a source of deformity; and as they are frequently found on the face, it may become desirable to remove them on this account, independently of the various mischief to which their increase might lead. A variety of methods have been proposed for dealing with these nævi, when it is wished to reduce their size or to take them away altogether. Mr. Abernethy has given some observations on this subject: he observed the circumstance of their being occasionally of a higher temperature than the rest of the surface of the body, and therefore thought that, if cold applications were used, so as to reduce the temperature, and then the part was subjected to pressure, they might be removed in that way. He mentions a case where an extensive nævus on the forearm of a child was thus reduced. It must be observed, however, that we generally have to treat those that are seated on the face and head, where pressure cannot well be applied : and, besides, pressure is sometimes found to irritate them, and make them grow faster, rather than to diminish them.

Excision is an obvious, and at all events an effectual mode of getting rid of these affections. In cutting them out, you must observe the rule of taking away the whole of the unnatural growth; you must cut into the sound parts all round ; you must not proceed economically, nor be inclined to save either skin or any other part in which any portion of this production may exist, because if you leave any thing behind, you have the growth reproduced, and if you cut into it in operating, you will have such profuse hæmorrhage that, unless you saw it, you would not think possible. You must cut freely in the circumference in the sound parts, so as to take away all the morbid growth; for although you have a good deal of hæmorrhage even in this way, yet you will not have that furious bleeding which results if you cut into the tumor. Very commonly, in extirpating them, you have a degree of hæmorrhage which makes you think for the moment that the child (if the operation be performed on an infant) will die from the loss of so much blood. I may mention that the operation of excision on this account is in some measure limited to nævi of a moderate size; for when the operation is performed on those of large size, there is really danger of immediate death under the knife, from hæmorrhage. Mr. Wardrop took away a large nævus from the back of an infant; he took it away as quickly as he could, but it was one of considerable size, and there was a most profuse bleeding from the whole of the divided surface, and in about one or two minutes the child was defunct. On coming to examine this nævus, which he had so removed, one of the vessels that was opened was found to be of caliber enough to admit the largest writing quill. When you consider that the whole surface, perhaps, is covered with these vessels, and see how freely they emit their blood, you cannot wonder at such an occurrence.

In order to avoid the danger of hæmorrhge from the use of the knife, it has been proposed to tie these tumors, to surround the base with a ligature, and draw it very tight, so as to produce mortification of the included part. In general the bases of these tumors are too large, and they are too little prominent to admit of surrounding them by a single ligature, but you pass under the base a strong needle with a double ligature, and when you cut off the needle you have two ligatures, which are to be tied one each way, so as to surround the base by two ligatures, each of which encloses one half. This is an effectual mode of preventing the danger of hemorrhage. You might suppose that when the ligature surrounds a nævus of considerable size, and includes a large mass of integument, that this mode of proceeding might be liable to much inconvenience. I have operated, however, in a considerable number of cases, where the nævus was of considerable size, and I have in no instance seen inconvenience arising from the employment of the ligature. I have used it in cases where the nævus was so large that I dared not to extirpate it with the knife, and the operation has been successful, so that the proceeding seems to be a very safe one.

It has been proposed in nævus, as in aneurism by anastamosis, to prevent the growth of the tumor, and to produce its reduction, by tying the arterial trunks that feed it. Now in general we find that the vessels seem to come into them from all quarters, so that it is very difficult to do this. However this has been more particularly attempted where the nævi have been seated upon the head, and where the carotid artery could be tied. Mr. Wardrop performed the operation of tying the carotid artery in a child for a large nævus on the head. This seemed to produce a considerable reduction in the size of the tumor; the child, however, was enfeebled and in a reduced state in consequence of partial ulceration and loss of blood from the nævus, and died more from that than from any other cause; so that in this case the effect of tying the artery could not be well estimated. In consequence of this case of Mr. Wardrop, tying the the carotid artery was tried by Dupuytren in a large nævus situated about the ear, and including the whole texture of the surrounding parts. By pressure on the carotid artery, it was found that the size of the nævus was reduced, and that it became flaccid. Hence the natural inference was, that if the artery were tied, the nævus would be reduced in size. Dupuytren tied the common carotid artery, and for a few days the effect of the operation seemed to correspond with the wishes of the operator; the tumor became flaccid, but it soon swelled in size again, and after a little time it grew as fast as ever; so that, in fact, according to the experience that we have at present, the plan of tying the arteries that go to these nævi, or the main trunks of the vessels which supply the part of the body in which they are situated, cannot be much relied on.

I remember a case of aneurism by anastomosis, seated on the finger of a young girl, on whom Mr. Hodgson operated. This was not a nævus; this was a production of the vascular kind, that came subsequently to birth, and formed a considerable swelling, occupying a great part of the finger, with a strong pulsation in it, and a degree of heat, not only in the finger, but in the whole hand. Mr. Hodgson tied the radial and ulnar arteries in that case, having first pressed upon them to see the effect, and having been satisfied that doing this materially reduced the tumor and stopped the pulsation. This operation was attended at the time with an interruption of the pulsation, and a reduction of the swelling; but the effect was only temporary, and, in fact, the girl suffered so much, that she wished me to do any other kind of operation that I could think of for her. I accordingly subsequently cut round the base of the tumor upon the finger; that is, I cut round the tumor between its basis and the hand, so as to divide the whole of the soft parts except the flexor and extensor tendons; and in doing this there was a pretty considerable hemorrhage; for, of course, there was a necessity that all the enlarged vessels should be divided. This had the effect, I will not say of removing the tumor, but certainly of arresting its growth. I may mention that a question was entertained, if the skin were cut circularly round, and all the soft parts were divided except the tendons, whether or not mortification would occur. However, when I divided the vessels (I may mention that one of the digital arteries was as large as the radial artery), the arteries at the distal extremity, coming from the point of the finger, bled so freely, that I was obliged to tie some of them. The circulation in the small vessels, along the periosteum, proceeded in such a manner as to be quite sufficient to sustain the vitality of the parts.

Then another principle of treatment for these nævi, were you do not like to extirpate them, or the application of the ligature may be impracticable (for sometimes the figure and situation of the nævus are such that a ligature cannot be applied,) consists in a kind of attempt to imitate the process of natural cure which I mentioned consist in an accession of inflammation and ulceration, sometimes leading to partial sloughing of the tumor. It is the application of caustic to the tumor. Mr. Wardrop recommends an application to the centre of the tumor of a portion of potass, or rubbing the tumor thoroughly with the nitrate of silver; but the potass is the most effectual. He says it will produce a certain degree of inflammation at one part of the tumor, like the natural process, and that this will diffuse itself over the whole of the texture; and thus you will get rid of a nævus which is too large to attack with the knife. Not long ago he sent me a patient to look at, with a large nævus on the neck, which he thought too large to admit of being treated by excision or ligature. He applied caustic in the way that I have mentioned. This cure is going on favorably; and he has employed it to another, which is also going on well. At all events, you know from this that the caustic may be safely employed in cases of this nature.

Another method lately proposed, and which in some instances has been adopted with great success, is that of vaccination on the nævus, in children who have not previously been the subject of the vaccine disease. You introduce the vaccine matter at points all over the nævus, and round it, so as to produce a considerable degree of inflammation in the textures. I have tried this in two or three instances: in one case with complete success; in one case with partial success; and I have seen an instance lately where it has entirely failed, although the nævus was not large, and I was obliged to extirpate it. It was seated on the scalp.—*Med. Gazette.*

---

### MIDWIFERY.

*Employment of Secale Cornutum in a Case of Hemorrhage from Inertia of the Uterus.* By M. P. L. Maurage. (*Archives*, Avril 1830.)

M. Maurage was called to a woman who had been delivered seven hours, after a natural but long and painful labour. The placenta was not expelled, and a violent hemorrhage, which had arisen immediately after the birth of the child, still continued, and produced frequent syncope. The patient's face was pale, and she appeared much exhausted: pulse sixty, but scarcely perceptible. Upon examining the abdomen, no projection of the uterus was discoverable. The belly was supple, and not painful. The cervix uteri easily permitted the introduction of the hand, and it was ascertained that the placenta adhered throughout nearly its whole extent. The uterus appeared in a state of complete relaxation. No contraction was produced by moving the hands and fingers about within its cavity, or by friction on the abdomen. Under these circumstances, it was apprehended that the hemorrhage would increase if the placenta were separated, from a want of uterine power, and it was determined to give the ergot.

M. M. withdrew his hand from the uterus, after having kept it in about fifteen minutes. Ten grains of coarsely-powdered ergot were infused in four ounces of sugar and water, and given at one dose. In fifteen minutes the hemorrhage was less, but the patient had experienced no pain. In one hour the hemorrhage was not half so great as it had been, but still the uterus did not contract. In two hours from the first dose of the remedy, the same quantity was repeated: and in twenty minutes more the hemorrhage ceased entirely, and contractions of the uterus were plainly felt through the abdominal muscles. After waiting an hour, attempts were made to extract the placenta, but the cervix uteri was so much contracted as scarcely to admit one or two fingers. The placenta still remained adherent to the uterus; but, as the hemorrhage had ceased, and there was no necessity for any immediate measures, M. M. waited still two hours longer, when, the pains having entirely subsided, and the natural separation of the placenta no longer probable, a third dose of the ergot was given. In fifteen minutes afterwards, the pains in the back returned, and in about an hour slight uterine contractions took place, which gradually expelled the placenta in the course of three or four hours. Its expulsion was not attended by any hemorrhage or severe pain, and the patient recovered perfectly without any unfavorable symptoms.

